# Glyphosate Use, Toxicity and Occurrence in Food

**DOI:** 10.3390/foods10112785

**Published:** 2021-11-12

**Authors:** Diogo Soares, Liliana Silva, Sofia Duarte, Angelina Pena, André Pereira

**Affiliations:** 1 LAQV, REQUIMTE, Laboratory of Bromatology and Pharmacognosy, Faculty of Pharmacy, University of Coimbra, Polo III, Azinhaga de Stª Comba, 3000-548 Coimbra, Portugal; daniguito97@hotmail.com (D.S.); ljgsilva@hotmail.com (L.S.); s.cancela.duarte@gmail.com (S.D.); apena@ci.uc.pt (A.P.); 2 Vasco da Gama Research Center, Escola Universitária Vasco da Gama, 3020-210 Coimbra, Portugal

**Keywords:** glyphosate, environmental contaminant, toxicity, public health, food safety

## Abstract

Glyphosate is a systemic, broad-spectrum and post-emergent herbicide. The use of glyphosate has grown in the last decades, and it is currently the most used herbicide worldwide. The rise of glyphosate consumption over the years also brought an increased concern about its possible toxicity and consequences for human health. However, a scientific community consensus does not exist at the present time, and glyphosate’s safety and health consequences are controversial. Since glyphosate is mainly applied in fields and can persist several months in the soil, concerns have been raised about the impact that its presence in food can cause in humans. Therefore, this work aims to review the glyphosate use, toxicity and occurrence in diverse food samples, which, in certain cases, occurs at violative levels. The incidence of glyphosate at levels above those legally allowed and the suspected toxic effects of this compound raise awareness regarding public health.

## 1. Introduction

Glyphosate is an organophosphorus herbicide [1]. The herbicide function of glyphosate was discovered in 1970 by John Franz, a chemist from Monsanto^®^ company (St. Louis, MO, USA), which produced several years later the first glyphosate-based herbicide (GBH), Roundup^®^ [2]. Nowadays, there are hundreds of GBHs commercialized under different brands in more than 100 countries across the world [3]. Currently, glyphosate is the most used herbicide worldwide [4].

The exponential rise in glyphosate use over the years also brought an increased concern about its possible toxicity and the eventual consequences to human health. Therefore, the number of studies about glyphosate effects on the human health increased in recent years [5]. Glyphosate is applied intensively in crop fields, and its residues are frequently detected in the environment, particularly in plants, soil, water, food products and also in human urine [6]. Consequently, concerns increased within the scientific community about the potential impact that this herbicide and its metabolites can have in the environment and humans. Hence, the commercialization of GBHs is highly regulated, and there are maximum residue limits (MRLs) established for glyphosate residues in foods.

This review aims to evaluate the sources and occurrence of glyphosate in different foods and its environmental and human health effects.

## 2. Methodology

A careful literature review was performed regarding data on the occurrence of glyphosate in food and other related subjects like sales, consumption or toxicity. Various platforms were used to retrieve the data for this review, including Google, Google Scholar, PubMed and Science Direct.

The extensive search to find relevant publications included the following keywords, individually and in combination: Glyphosate, Consumption, Sales, RoundUp, Properties, World, Europe, Agriculture, Glyphosate applied in fields, Herbicide, Sales, Toxicokinetics, Absorption, Distribution, Metabolism, Excretion, Health Impact, Toxicity, Side Effects, Acute Toxicity, Residues, Chronic Toxicity, Cytotoxicity, Carcinogenicity, Cancer neurotoxicity, Organ damage, Carcinogen, Genotoxicity, Teratogenicity, Endocrine disruption, Environmental Impact, Environment, Legislation, Maximum Residue Levels, Methodologies, Analytical Methodologies, Food, Occurrence.

Only the publications from 2000 onwards were considered. Publications sponsored by, or with authors affiliated to, the herbicides industry were excluded. Publications regarding analytical methodologies that did not present detection or quantification limits were excluded.

To obtain the data on glyphosate consumption, besides international publications, some websites like Eurostat and national institutes from the European Union were consulted. Regarding the legislation, the data were collected from the European Union’s legislation database.

## 3. Physical and Chemical Properties

Glyphosate is a herbicide that belongs to the family of organophosphorus compounds [1]. Currently, glyphosate is widely applied in fields due to its herbicidal properties. However, those properties were not discovered when glyphosate was synthetized for the first time in 1950, being only patented several decades later [7].

**Figure 1:**
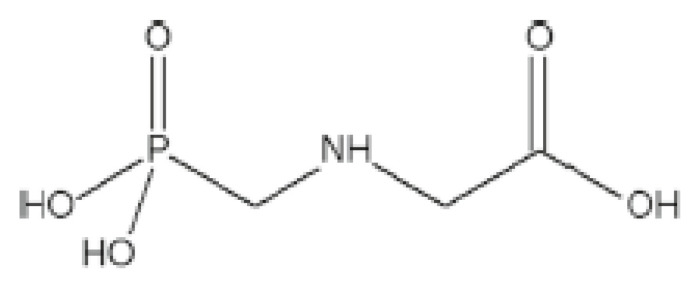
Glyphosate chemical structure.

Regarding its chemical structure (Figure 1), glyphosate is a zwiterrion [8] with phosphonate, carboxylate and amine functions. The zwitterionic structure of glyphosate affords the ability to chelate with trivalent and quadrivalent metals [9,10,11].

The covalent bond between the carbon and the phosphorus atoms is characteristic of these organophosphate compounds and provides glyphosate with several chemical and physical specificities, such as high adsorption, high water solubility and compatibility with other chemical substances [9].

Glyphosate is a molecule with high polarity, contributing to its high solubility in water and insolubility in organic solvents [3]. The particular physical and chemical properties of glyphosate (Table 1), such as the absence of a chromophore or a fluorophore group, the non-existence of absorption in the ultraviolet region, its low ionization, low volatility and high hydrophilicity [12], demand the use of complex analytical methodologies for the detection and quantification of this herbicide in order to achieve the sensitivity and accuracy requested [6,8,13,14].

## 4. Glyphosate-Based Herbicides

In its acid form, glyphosate is less soluble than in its salt form. Therefore, the GBHs consist of glyphosate in its salt form, namely isopropylamine, ammonium, sodium, potassium and trimethylsulfonium. Among all these glyphosate salts, isopropylamine is the most used in agriculture [10]. In addition to glyphosate, GBHs contain polar surfactants, such as polyoxyethyleneamide (POEA), sulfuric acid and phosphoric acid [15,16]. These will enhance the herbicidal action of glyphosate by increasing its solubility in water as well as promoting its penetration and absorption in the plant [2,9].

GBHs must have a minimum glyphosate purity of about 950 g/kg [14], and the impurities most frequently found are formaldehyde (with a maximum amount of 1.3 g/kg), N-nitrosoglyphosate (with a maximum amount of 1 mg/kg) and N-nitrous-phosphonomethylglycine [16,17]. Nowadays, hundreds of GBHs are registered under different commercial brands in more than 100 countries worldwide [3].

### 4.1. Sales and Use

#### 4.1.1. Worldwide

**Figure 2:**
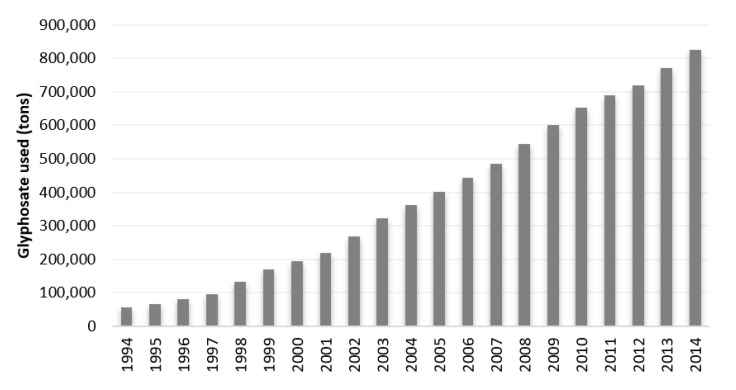
Evolution of annual glyphosate consumption worldwide between 1994 and 2014.

In 1974, its first year of commercialization, the consumption of glyphosate was about 3 thousand tons. Since then, the annual consumption of glyphosate (Figure 2) has increased exponentially, from about 56 thousand tons in 1994 to more than 825 thousand tons in 2014 [4]. Unfortunately, no data regarding the last few years were found. This exponential increase makes it the most widely used herbicide globally [18,19], being widely used in agricultural production in both developed and developing countries [15]. Estimates show that the annual consumption of glyphosate will continue to increase, and it is expected that in the next few years the milestone of 1 million tons of glyphosate used worldwide will be reached [16].

#### 4.1.2. European Union

Concerning the consumption of glyphosate in the EU, the information available is limited. A request was made electronically to Europe Direct (EDCC) and the European Statistical Office (EUROSTAT) for data on glyphosate consumption in the EU, but they reported that they did not have these data. The latest data available from the EU, that date back to 2003 [20], are shown in Table 2. It appears that in that year glyphosate was the most widely consumed herbicide in the EU. However, the quantity consumed was given as confidential.

**Figure 3:**
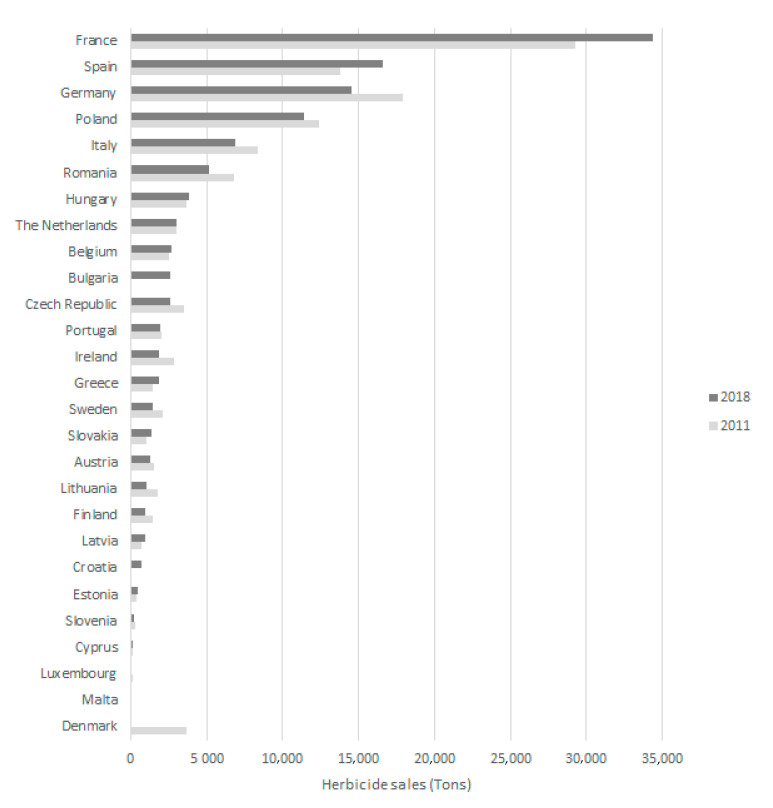
Herbicide sales in 2011 and 2018 in the EU.

The EU updates data on the MS consumption of pesticides, including total herbicides, every year, through EUROSTAT (Table S1, Supporting Information) [21]. Accordingly, Figure 3 presents the herbicide sales in 2011 and 2018. All countries shared data on herbicide consumption, except Bulgaria and Croatia, in 2011, and Denmark, in 2018. It can be observed that in 2018 the leading country for herbicide use in the EU was France, with around 35,000 tons, followed by Spain, with more than 16,000 tons, and Germany, with sales higher than 14,000 tons. On the other hand, the three countries with the lowest consumption of herbicides, in 2018, were Malta, Luxembourg and Cyprus.

Regarding the herbicide sales evolution between 2011 and 2018, it can be seen that in the vast majority of countries there was a decrease, with Malta, Luxembourg and Lithuania standing out with decreases of 48%, 47% and 41%, respectively. On the other hand, in the same period, there was an increase in herbicide sales in Latvia, Greece, Slovakia, Estonia, Spain and France of 34%, 26%, 23%, 20% and 18%, respectively.

**Figure 4:**
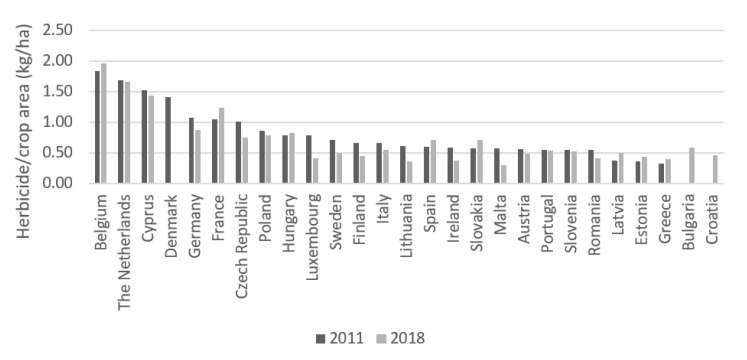
Herbicide use intensity (kg/ha) in the EU between 2011 and 2018.

However, annual herbicide sales in each EU MS are not an indicator of the intensity of herbicide use in each country. The concept of herbicide applied per agricultural area is one of the most important parameters for determining the intensity of a herbicide use [4]. Thus, in Figure 4 (Table S2, Supporting Information), it appears that the countries that applied most herbicides per hectare of agricultural area (kg/ha) on their agricultural land in 2011 were Belgium, the Netherlands and Cyprus, with 1.83 kg/ha, 1.68 kg/ha and 1.52 kg/ha, respectively. In 2018, these same countries continued to be the most intensive users of herbicides, with Belgium increasing to 1.96 kg/ha in contrast to the Netherlands and Cyprus, which decreased to 1.66 kg/ha and 1.43 kg/ha, respectively. On the other hand, the countries with the lowest use of herbicides on their agricultural land in 2011 were Latvia, Estonia and Greece, with 0.37 kg/ha, 0.36 kg/ha and 0.32 kg/ha, respectively. In 2018, Ireland, Lithuania and Malta applied 0.38 kg/ha, 0.36 kg/ha and 0.30 kg/ha, respectively [21,22].

These variations in herbicide consumption levels do not reflect the variation in glyphosate consumption in that same period. Therefore, since the EU did not have this data, research on glyphosate consumption was conducted within each MS (websites of parliaments, ministries associated with the environment and agriculture and national statistical platforms, among others). The vast majority of MS do not provide data on the consumption of each herbicide, only the annual consumption of total herbicides (as the EU itself). Only Germany [23,24], Belgium [25], Estonia (although with some confidential data) [26], Denmark [27], Czech Republic [28,29,30,31,32,33,34], France [35] and Portugal [36,37,38,39,40,41,42] provide information about the annual consumption of glyphosate (Table S3, Supporting Information).

**Figure 5:**
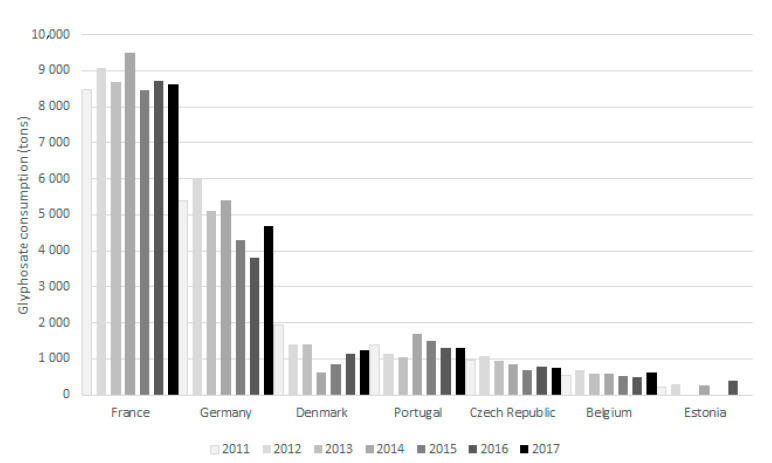
Glyphosate consumption in some European countries between 2011 and 2017.

Figure 5 shows the data collected on glyphosate consumption in some European countries between 2011 and 2017. Between 2011 and 2017, France was the country that used the highest amount of glyphosate, followed by Germany. On the other hand, Estonia and Belgium presented a lower glyphosate consumption. With regard to the evolution of consumption in 2011 and 2017, there is generally a large fluctuation, year after year, in all countries. However, it can be observed that the use of glyphosate in 2017, comparing to 2011, decreased in Germany, Denmark, Portugal and the Czech Republic and increased in France and Belgium.

**Figure 6:**
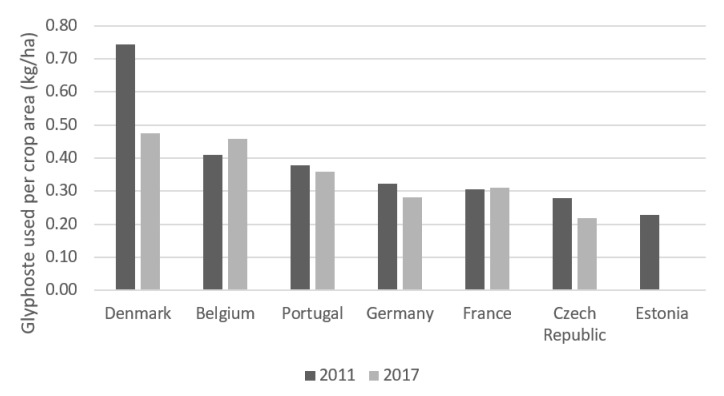
Evolution of glyphosate use (kg/ha) in some European countries, between 2011 and 2017.

The amount of glyphosate used per agricultural area makes it possible to determine the intensity of use of this herbicide in the different countries (Table S4, Supporting Information). Figure 6 shows that in 2011, the country with the highest use of glyphosate on its agricultural land was Denmark with 0.74 kg/ha while Estonia was the country with the lowest use, with 0.23 kg/ha. As for 2017, Denmark continued to be the leader with 0.47 kg/ha, while the Czech Republic was the country that applied the least, with 0.22 kg/ha.

Comparing the percentage of glyphosate sales with the total herbicide sales over the years in the EU, it can be concluded that it has an increasing share, thus consolidating its status as the best-selling herbicide in the country throughout the 21st century.

### 4.2. Action Mechanism in Plants

From the point of view of its action mechanism, glyphosate is a systemic, non-selective and post-emerging herbicide [7,43,44]. A herbicide is systemic when it is absorbed through the plant, followed by translocation through it [3]. A herbicide is non-selective and post-emerging when it acts either on weeds or on grass that has already germinated [45].

Glyphosate acts by inhibiting the shikimate pathway—more specifically, by inhibiting the 5-enolpyruvylshikimate-3-phosphate synthase (EPSPS). With the EPSPS inhibition, the synthesis of tyrosine, tryptophan and phenylalanine, essential amino acids for plant growth, is blocked [7,23]. Glyphosate, the only herbicide that inhibits EPSPS, also compromises the production of secondary metabolites, such as lignin [11].

## 5. Toxicokinetics in Humans

### 5.1. Absorption

Studies in rats show that when administered orally, glyphosate has a rapid but incomplete absorption, with only about 20 to 30% of the administered dose being absorbed [46]. Another study shows that oral absorption is lower when a higher dose of glyphosate is administered [3].

The skin absorption of glyphosate is limited, with only about 1 to 3% of this herbicide being absorbed [46].

### 5.2. Distribution and Metabolism

Only 1% of the absorbed dose of glyphosate remains in the rat’s body after 7 days, which demonstrates that it does not accumulate in the body. It has been demonstrated that glyphosate does not undergo enterohepatic circulation [14]. The highest concentrations of glyphosate in the body have been detected in the small intestine, liver, kidneys and bones [3,14,46].

Glyphosate is poorly metabolized both in plants and animals [47]. It is excreted mostly unchanged, and only about 1% undergoes metabolism, via hydrolysis, originating aminomethylphosphonic acid (AMPA), the main metabolite of glyphosate [43,46].

### 5.3. Excretion

Feces are the main route for rats’ glyphosate elimination, and about 60 to 70% of the administered dose is eliminated by this route [3]. The remaining 20 to 30% are rapidly eliminated by the urinary route [46,48]. The excretion via the bile and lungs is residual [46].

It is estimated that glyphosate’s half-life is between 6 and 12 h. The great majority of glyphosate and its metabolites are excreted after 48 h, and after 7 days practically all of them have been eliminated from the body [14,46].

## 6. Human Health Impact

The increase in glyphosate consumption over the years has also brought increased concerns about the possible toxicity effects of this herbicide and possible consequences for human health. Therefore, in recent years, studies on the effects of glyphosate on human health have increased. This discussion has a major drawback, which is the fact that some toxicity studies have the participation of the herbicide industry, which has a commercial interest in maintaining the authorization of its best-selling herbicide. Nonetheless, there is currently no consensus among the scientific community, and there is controversy over the safety of glyphosate and its health consequences.

### 6.1. Toxicological Parameters

Due to increased concerns about glyphosate’s toxicity, the European Food Safety Authority (EFSA) carried out in 2015 a review on the risk associated with the use of glyphosate, and the following toxicological endpoints were defined or reviewed based on laboratory studies in rabbits (EFSA, 2016):

- No Observable Adverse Effect Level (NOAEL) of 100 mg/kg body weight per day.
- Acceptable Daily Intake (ADI) of 0.5 mg/kg of body weight per day.
- Acute Reference Dose (ARfD) of 0.5 mg/kg of body weight per day.
- Acceptable Operator Exposure Level (AOEL) of 0.1 mg/kg body weight per day.

### 6.2. Acute Toxicity

The measure of the acute toxicity of a substance is the lethal dose to 50% of the population (LD_50_), which corresponds to the dose required for a given substance to kill 50% of the population tested.

Through several experimental studies in rats, several institutions have determined the LD_50_ for the oral and dermal pathways. At the European level, EFSA defined, in 2015, a LD_50_ of more than 2000 mg/kg of body weight for both the oral and dermal pathways [14]. Worldwide, the Food and Agriculture Organization (FAO) and the World Health Organization (WHO), in a joint opinion, defined, in 2016, a LD_50_ of 5600 mg/kg of body weight for the oral pathway and more than 2000 mg/kg of body weight for the dermal pathway [46].

Since glyphosate is sprayed on agricultural fields [15], another useful measure is the lethal concentration for 50% of the population (LC_50_), which corresponds to the concentration of a given substance in the air that, for a given time, causes the death of 50% of the study population. While EFSA has defined a LC_50_ greater than 5 mg/L of air for an exposure period of 4 h [14], FAO has defined a LC_50_ greater than 5.46 mg/L of air for the same exposure period [46].

According to the acute toxicity classification used in the United States, glyphosate is classified in category IV as a practically non-toxic substance [3]. Observational studies carried out on workers who applied GBHs show that glyphosate causes severe eye irritation and moderate skin irritation [14,46]. The European Chemicals Agency (ECHA) classifies glyphosate as an eye irritant, as it causes serious eye damage [49]. This could be observed in rabbits at exposures above 65 mg [49].

Additionally, zebra fish toxicity studies also reported cardiotoxicity (48-h study) and increased mortality and malformation at a concentration of 8.5 mg/L (72 h), presenting an LD50 of 66.04 mg/L (48-h study) [50,51]. Cardiotoxicity was observed even at lower concentrations (µg/L) (72h study). However, comparing glyphosate and AMPA, a higher toxicity was observed for AMPA [52].

Cases of acute toxicity in humans were detected after the accidental or intentional ingestion of GBHs, leading to weight loss, gastrointestinal, pulmonary, renal and liver disorders [46,53].

### 6.3. Chronic Toxicity

#### 6.3.1. Target Organ Toxicity

Several studies have been conducted in recent years to evaluate the toxicity of glyphosate and GBHs in target organs. One study revealed that the exposure to glyphosate is associated with gastrointestinal problems, including an increased risk of celiac disease [54]. Additionally, studies have demonstrated the cardiotoxic effects of glyphosate in humans through the detection of anomalies in the electrocardiogram, namely an extension of the QT segment and arrhythmias after repeated exposure to concentrated doses of GBHs [55,56].

Several studies have also shown that glyphosate and GBHs can cause oxidative stress and damage certain organs, particularly the liver, due to increased oxygen free radicals [17,57]. In 2017, a study in rats showed that chronic exposure to low concentrations of GBHs has hepatotoxic effects. Changes in proteome and hepatic metabolome were found, demonstrating an overlap with the biomarkers of non-alcoholic fatty liver disease and its evolution to non-alcoholic steatohepatitis. This proves a hepatic dysfunction associated with the exposure to GBHs [58].

In 2019, a study in rats showed that the chronic exposure to GBHs is nephrotoxic, leading to the loss of tubular cells by apoptosis [59]. Furthermore, several cases of chronic renal disease of unknown etiology have appeared, in recent years, in areas of Sri Lanka where there was intensive use of GBHs. Glyphosate is suspected to be the possible cause of this chronic kidney disease, but no study has yet demonstrated this association [60].

However, the last report on glyphosate, published by ECHA in 2017, stated that glyphosate was not toxic to target organs in humans [49].

#### 6.3.2. Cytotoxicity

Recently, studies have been performed with human cells to evaluate the cytotoxicity of glyphosate and GBHs. A study conducted with human erythrocytes showed that GBHs caused morphological changes in these cells [61]. Another study conducted with liver, lung and nerve cells demonstrated that there is a risk of cytotoxicity associated with GBHs, but this risk may not be directly related to glyphosate but to the other constituents of GBHs [62].

#### 6.3.3. Carcinogenicity

In recent years, several government agencies as well as international agencies have performed an evaluation of glyphosate’s carcinogenicity. However, this evaluation does not meet with the consensus of the scientific community, and there is, nowadays, a huge controversy regarding the status of glyphosate as a carcinogen. The differences in results between different agencies may result from differences in data collection (inclusion or exclusion of certain scientific studies), methods of analysis and interpretation of results that may be ambiguous [5].

In 2015, IARC classified glyphosate in group 2A, i.e., probable human carcinogen. This classification is based on insufficient evidence of carcinogenicity in humans (studies have shown a positive association between glyphosate and non-Hodgkin lymphoma (NHL)) and satisfactory evidence in animal clinical studies [17,63]. This IARC conclusion was strongly criticized by the scientific community due to the absence of concrete evidence of carcinogenicity in humans.

In the same year, EFSA published a report highlighting the absence of a clear association between glyphosate and cancer in humans, culminating in the absence of the need to classify glyphosate in relation to carcinogenicity [14]. In 2016, FAO and WHO issued a joint report on pesticide residues in food where they concluded that it is unlikely that humans exposed to glyphosate through diet will develop a process of carcinogenesis, and therefore that glyphosate is not a carcinogen [46]. One year later, in 2017, ECHA, like EFSA, concluded that glyphosate was not a carcinogen [49].

As for the government agencies, the Commission for Food Security of Japan in 2016 [53], the Australian Pesticides and Veterinary Medicines Authority in 2017 [64] and the Environmental Protection Agency of the United States of America (USA) in 2019 [65] also concluded that glyphosate is not carcinogenic. However, in 2019, the Agency for Toxic Substances and Disease Registry (ATSDR) from the USA, in a report on glyphosate’s toxicity, concluded, as did IARC, that there is a potential cancer risk associated with the use of glyphosate and GBHs [66,67].

Regarding recent studies, a meta-analysis published in 2019 showed an increased risk of NHL in individuals heavily exposed to GBHs [68], while a review of epidemiological studies published in 2020 reveals an absence of association between glyphosate exposure and the occurrence of NHL [69].

#### 6.3.4. Neurotoxicity

In vitro studies in human cells have shown that a low daily exposure to glyphosate can compromise the functioning of acetylcholinesterase, leading to deregulation in the transmission of nerve impulses and the consequent appearance of neurological disorders [15]. However, animal studies show an absence of neurotoxicity even at high concentrations, so several institutions, including EFSA and FAO, do not consider glyphosate a neurotoxic substance [14,46,53].

#### 6.3.5. Genotoxicity

In 2015, IARC classified glyphosate as a genotoxic agent based on studies that showed that glyphosate and GBHs caused damage to mammalian chromosomes and deoxyribonucleic acid (DNA) and human cells in vitro [17,63]. In the following years, several international institutions, including EFSA and FAO, published reports on glyphosate’s toxicity where it was shown that glyphosate had no genotoxic potential in humans [14,46].

In order to evaluate the genotoxic potential of glyphosate, several studies have been conducted in recent years. In 2017, an in vitro study showed that glyphosate could induce DNA damage in human leukocytes and epigenetic changes in animal cells [70]. A systematic review, published in 2019, reveals that there is a genotoxic effect associated with exposure to GBHs. However, genotoxicity may not be directly associated with glyphosate, but with POEA, a surfactant present in GBHs [2].

#### 6.3.6. Teratogenic Effects

Several epidemiological studies conducted in several South American countries have reported an increasing number of malformations in fetuses in areas of high application of GBH, thus highlighting the teratogenic potential of this herbicide [71]. However, in 2016 the FAO concluded in its report on pesticides that glyphosate is not teratogenic. This conclusion is based on several studies carried out on rats, in which no teratogenic effects were found with daily doses of up to 3500 mg of glyphosate per kilogram of body weight [46].

#### 6.3.7. Endocrine Disruption

Currently, no state or international institution has included glyphosate in the list of endocrine disrupters due to the lack of studies that show an interaction of glyphosate with the endocrine system in mammals [14]. However, studies with human and animal cells have shown that long exposures to low doses of glyphosate and GBHs may cause endocrine system disorders [57].

Although several entities do not consider glyphosate as toxic, several studies report toxicity effects, so until there is clarification on this subject the precautionary principle should prevail.

## 7. Environmental Impact

Since glyphosate is mainly applied in agricultural fields, and due to the increasing consumption of glyphosate in recent decades, the concerns about the impact that glyphosate and its metabolites may have on the environment have grown [68].

Glyphosate is degraded in the environment, particularly in soils, by bacteria through two pathways. The predominant pathway results in the formation of the main glyphosate metabolite, the AMPA, by the action of glyphosate oxiredutase. However, the decomposition of glyphosate also occurs in the plants themselves, therefore, glyphosate and AMPA residues can also be found in plant products [15].

Depending on the climate and the soil where GBHS are applied, residues of both glyphosate and AMPA can persist in the soil for up to approximately 6 months [72]. The fact that glyphosate persists for several months in the environment can impact ecosystems [16]. A study revealed that glyphosate causes structural changes in the microbial population of soils, causing the development of phytopathogenic fungi [11]. Another study concluded that glyphosate had a direct impact on the morphology and reproduction of several species of worms [9].

On the other hand, glyphosate has been found to have the capacity to contaminate aquatic ecosystems [54], which resulted in ECHA classifying glyphosate as toxic to aquatic life with persistent effects in 2017 [49].

## 8. Legislation and Maximum Residue Levels in Food

The growth in the consumption of GBHs in recent decades has brought concerns about the possible toxicity of glyphosate and of these formulations. In 2016, the renewal of the marketing authorization for GBHS was debated in the European Parliament (EP), and this resolution was rejected [73]. In 2017, the European Commission (EC) revoked the decision taken previously by the EP and decided to renew the approval of the sale of glyphosate in the European Union (EU) for a period of 5 years, until December 2022. However, due to increasing concerns over the safety of POEA, a surfactant present in several GBHs, the EC has banned the commercialization of GBHs containing this co-formulant in all its Member States (MS) [74].

The MRL corresponds to the maximum legally permitted amount of residues of a given contaminant in food for human consumption. In the EU, the EC is responsible for setting the MRLs allowed in foodstuffs (Table S5, Supporting Information), that vary between 20,000 μg/kg in oat cereals and 0.1 μg/L in water [75].

In 2019, at the request of the EC, a review of the glyphosate MRL was carried out by EFSA, the highest authority for food safety at the European level [76]. However, although EFSA has already published this review, to date the MRLs for glyphosate have not been updated by the EC [77].

## 9. Analytical Methodologies

The physicochemical characteristics of glyphosate, namely low molecular weight, high polarity, absence of ultraviolet absorption, high solubility in water, low ionization and low volatility, make it a compound difficult to detect with conventional analytical methods [12,13,78]. On the other hand, the absence of a chromophore group in the glyphosate structure makes it difficult to detect it directly through chromatography coupled to a photometer, and it is necessary to use derivatization to increase the sensitivity of the method [10]. Thus, numerous alternative analytical methodologies have been developed to detect and quantify glyphosate in food (Table 3).

The limit of detection (LOD) and the limit of quantification (LOQ) are two fundamental parameters for evaluating the sensitivity of an analytical method. An adequate analytical methodology for the detection of glyphosate in food is one that has values of LOD and LOQ well below the MRLs.

Currently, the method that has the highest sensitivity and selectivity for the evaluation of glyphosate in food is HPLC-MS/MS, and it is also the method recommended by the European Union Reference Laboratory for Pesticide Residues [80]. However, there are other methodologies that present good sensitivity, namely UHPLC-MS/MS, already applied in vegetables and fruits, with a LOQ of 3 μg/kg, and FASI-MEKS, used in cereals analysis, with a LOQ of 100 μg/kg. These values are about 30 and 100 times lower than the MRLs defined, respectively. On the other hand, the use of IC-HRMS in foods of animal origin and of FI-MS in fruits and vegetables is not adequate, since these methodologies present LOQs superior to the defined MRLs.

## 10. Occurrence in Food

As already mentioned, the increase in glyphosate consumption in recent decades has raised concerns by the scientific community about the impact it can have on human health. Thus, studies have been conducted in several countries to assess human exposure to glyphosate through the analysis of different food categories.

### 10.1. Olive Oil

Since Spain is one of the world’s largest producers of olives and olive oil, a study was carried out in Almería, southern Spain, to evaluate the glyphosate existing in different types of olive oil and oils, certifying that the levels of glyphosate complied with the MRL of 100 μg/kg defined by the EC [77]. In a total of 25 samples analyzed, no glyphosate residues were detected in any of the samples (the analytical method used had a LOD of 3.3 μg/kg) [43].

### 10.2. Honey

The application of glyphosate in agricultural fields can lead to the deposition of residues of this herbicide in the environment, particularly in flowers. In addition to bees being pollinators, insects are also honey producers through the collection of nectar from flowers. Thus, several studies were conducted to evaluate glyphosate in honey samples (Table 4).

Studies in Canada [19] and Switzerland [47] detected the presence of glyphosate in almost all samples, but at values below the MRL of 50 μg/kg [77]. In the Estonian study, although glyphosate was detected in a small number of samples, there were two samples that contained glyphosate levels above the MRL up to 62 μg/kg [79]. In the USA, residues were detected in about 30% of the samples, more than half at levels that were much higher than the MRL, including a sample that was seven times higher than allowed (342 μg/kg) [92]. On the other hand, a multinational study conducted by EFSA revealed that in 186 honey samples, 24 contained glyphosate, 8 of which were higher than legally permitted [93].

### 10.3. Fruits and Nuts

Several studies have been conducted to evaluate glyphosate in fruit and nut samples (Table 5).

In France, six samples were analyzed and no glyphosate residues were detected in any of the samples [91]. Another study, conducted in China, detected the presence of glyphosate in a pear sample, but in values below the MRL of 100 μg/kg [77,84]. In a Swiss study, all the fruit juice samples analyzed contained glyphosate up to 3.5 μg/kg, but no sample exceeded the permitted MRL [47].

A multinational study conducted by EFSA, in which a large number of samples of different types of fruit were analyzed, revealed the presence of glyphosate in a small number of samples, with only one pear sample having values higher than legally allowed [93].

In Portugal, DGAV is the authority responsible for controlling pesticide residues in food [44]. The last published report, referring to the year 2017, reveals that in all the products of vegetable origin tested, no glyphosate residues were detected and, consequently, the glyphosate MRL was not exceeded [94].

### 10.4. Cereals and Cereal Products

The application of glyphosate in agricultural fields where cereals are grown can lead to the accumulation of residues of this herbicide in the soil and cereals. In this way, several studies have determined the levels of glyphosate in several types of cereals as well as in cereal-based foods (Table 6).

A study conducted in Switzerland detected the presence of glyphosate residues in several samples, with about 90% of wheat samples, 80% of breakfast cereal samples and 70% of bread samples having glyphosate residues. Some samples contained glyphosate values above the MRL, namely one sample of bread with values four times higher than legally allowed and three samples of breakfast cereals with values up to 29 times higher than the MRL of 10 μg/kg defined by the EC [47,77]. Samples of breakfast cereals analyzed in a French study also contained higher levels than legally allowed, up to 34 μg/kg [91].

Another study conducted in Italy has detected levels of glyphosate about 25 times higher than the legally allowed value of 10,000 μg/kg in one wheat seed sample [77,86]. In a multinational study conducted by EFSA in 2017, several samples of the main cereals grown in Europe were analyzed. The results revealed that there were glyphosate residues in a low percentage of samples, with six samples of rye, four of pseudo cereals and one of rice exhibiting levels (243,000 μg/kg) that exceeded the MRL [93].

### 10.5. Vegetables

In recent years, several countries have conducted studies to evaluate glyphosate levels in vegetables and pulses (Table 7).

Studies from France [91] and China [84] have not detected the presence of glyphosate in several vegetables. In Ghana, 68 yam samples were analyzed, and 14 presented glyphosate residues, but at levels below the LOQ [13]. In the Swiss study, one third of the analyzed samples contained glyphosate residues, but below the MRLs of 100 μg/kg and 500 μg/kg [77] defined for vegetables and potatoes, respectively, at mean levels of 1.3 μg/kg [47].

On the other hand, another study carried out in Italy [82], as well as a multinational study carried out by EFSA [93], detected glyphosate residues above the legally permitted value in two canned vegetable samples and one asparagus sample, respectively.

A study in Switzerland [47] detected the presence of glyphosate residues in about half of the analyzed legume samples, but none of the samples exceeded the MRL of 10,000 μg/kg set by the EC [77]. A multinational study conducted by EFSA in 2017 [93] in samples of dried lentils, beans and soybeans also detected the presence of glyphosate in several samples, but with values below the legal limit.

### 10.6. Animal-Derived Products

Due to the exponential increase in the use of glyphosate in agriculture in recent decades, a study carried out in Switzerland [47] aimed to detect and quantify existing glyphosate residues in different samples of animal products (Table 8).

The results, ranging between <1 and 4.9 μg/kg, showed the presence of glyphosate residues in 23.1% of the meat and fish samples, but none showed values above the MRL of 50 μg/kg established [77].

### 10.7. Baby Food

Baby food has also been analyzed in several studies (Table 9). Studies in France [91] and Switzerland [47] did not detect the presence of glyphosate, while an Italian study detected the presence of glyphosate in 2 samples, but none had levels above the MRL of 10 μg/kg defined [93].

### 10.8. Water

In recent years, studies have been conducted to assess the presence of glyphosate in water (Table 10).

A study in Switzerland did not detect the presence of glyphosate in the surface water samples analyzed [95]. Another study, conducted in Mexico, detected the presence of glyphosate in practically all of the water samples analyzed, all of which had values (up to 0.78 μg/L) much higher than those legally allowed [96]. A German study also revealed the presence of glyphosate in 23 of the 39 samples analyzed. Of these, 10 contained glyphosate residues, in mean levels of 0.12 μg/L, above the MRL (0.1 μg/L) [97,100].

Another study conducted in the United States, involving several types of water samples, detected the presence of glyphosate in 1470 of the 3732 samples analyzed. One sample had values about 5000 times higher than the legally allowed value [98]. In a European study, thousands of surface water and groundwater samples from several countries were analyzed. Glyphosate residues were detected in about 30% of surface water samples. In 80% of these samples, the values were much higher than the MRL, including a sample that was 500 times higher than allowed. Only 1% of the groundwater samples contained glyphosate, of which more than half had values that exceeded the MRL, including a sample with 24 μg/L, a value 240 times above the limit [99].

### 10.9. Alcoholic Beverages

Although the EC does not define MRLs in wine and beer [77], studies have been conducted to evaluate glyphosate in these alcoholic beverages (Table 11).

A study conducted in Switzerland revealed the presence of glyphosate residues in all the wine samples analyzed, up to a maximum of 18.9 μg/L, and the presence of glyphosate in 2 of the 15 beer samples [47]. Although there is no MRL in wine, we can, for data analysis purposes, take as reference the MRL for water, which is 0.1 μg/L, and verify that all samples detected exceeded this value [100].

Another study conducted in Latvia analyzed the levels of glyphosate in 100 beer samples. The results revealed the presence of residues of this herbicide in 92 samples, with one sample showing glyphosate levels of 150 μg/L. Taking into account the MRL of the water, we found that all positive samples significantly exceeded this value [90].

Given the results of the studies, it is concluded that it is urgent to establish an MRL for alcoholic beverages.

In general, glyphosate residues are often detected in various food groups. Although, in the vast majority of cases, the values detected are within the legally allowed values, there are food groups where the MRLs were exceeded. In descending order of frequency of detection, these are water, honey, cereals and cereal products and vegetables. Regarding the values detected, the food group that generates the greatest concern is water, since it is the one with higher values in comparison to the MRL, and several samples are up to 5000 times higher than allowed.

## 11. Conclusions

It can be seen worldwide that in the last decades there has been a growth in the use of glyphosate. At the European level, there are only data on the consumption of herbicides in the EU, and there is no information about the consumption of glyphosate alone, which prevents us from verifying the accurate evolution of its consumption.

On the other hand, the increase in glyphosate consumption over the years has also brought an increased concern about the possible toxicity of this herbicide and the possible consequences for human health. Currently, there is no consensus in the scientific community about the toxicity of glyphosate, in particular regarding the possible carcinogenic potential of this herbicide. Therefore, further independent studies are needed to evaluate the toxicity of glyphosate as an active substance—especially studies on GBHs, as there may be constituents of the formulation that are toxic to humans. In addition to the human health impact, environmental concerns are increasing. The possible environmental impact that glyphosate may have is being questioned, and several studies have shown that both glyphosate and its metabolites have the capacity to accumulate in soils as well as contaminate aquatic ecosystems.

The EC is responsible for setting the MRLs allowed in food in Europe, and these limits are periodically reviewed. EFSA carried out a review of the MRL for glyphosate in 2019 at the request of the EC, but so far it appears that the EC has not adopted these values, so it is essential to do so as soon as possible. Otherwise, the national control reports on pesticide residues from each MS will not adequately identify samples that may pose a risk to public health.

Finally, several studies have been conducted in several countries to assess human exposure to glyphosate through food. Glyphosate residues have been detected in a large number of samples, sometimes in values that exceed the legally permitted limits, which can put at risk the most vulnerable populations, such as children and the elderly. It is essential to increase the number of studies as well as the number of samples analyzed in each study in order to have an accurate picture on glyphosate residues in food.

## Figures and Tables

**Table 1:** Glyphosate’s physical and chemical properties.

Active Substance	Glyphosate
Family	Organophosphorus compounds
Function	Herbicide
IUPAC name	N-(phosphonomethyl) glycine
CAS number	1071-83-6
Molecular formula	C3H8NO5P
Molecular weight	169.1 g/mol
Solubility	In water: 10.5 g/L a 20 °C Insoluble in organic solvents
Melting point (°C)	189 °C
Boiling point (°C)	Not defined (Glyphosate is decomposed during melting process)
Temperature of decomposition (°C)	200 °C
Dissociation constant	pKa1 = 2.0; pKa2 = 2.6; pKa3 = 5.6; pKa4 = 10.6
Log Kow	−0.40

**Table 2:** Top 10 most used herbicides in the EU in 2003.

	Herbicide	Quantity (in Tons)	Market Share (%)
1	Glyphosate	^1^	^1^
2	Isoproturon	12,073	14.3
3	MCPA	5293	6.3
4	Pendimethaline	3141	3.7
5	2,4-D	^1^	^1^
6	Trifluraline	2899	3.4
7	Acetochlor	2332	2.8
8	S-Metalachlor	^1^	^1^
9	Atrazine	1885	2.2
10	Metazachlor	1740	2.1

^1^ Confidential.

**Table 3:** Analytical methods used in glyphosate detection and quantification.

Matrix	Extraction Method	Analytical Method	LOD (μg/kg)	LOQ (μg/kg)	References
Honey Fish Beef	Sonication with a mixture of acidified water at 1% and methanol (7:3)	IC-HRMS	nd	43 51 65	[6]
Honey	SPE followed by derivatization	HPLC-MS/MS	nd	1	[19]
Honey	Centrifugation with methanol	UHPLC-MS/MS	nd	50	[79]
Yam	Centrifugation followed by derivatization	HPLC-MS/MS	40	120	[13]
Yam Grape Chickpea	SPE	FI-MS/MS	nd	500 500 2000	[80]
Fruits and Vegetables	Centrifugation with a mixture of water and methanol (1:1)	IC-MS/MS	25	nd	[81]
Fruits juice Vegetables Fruit puree	Centrifugation with acidified methanol	UHPLC-MS/MS	nd	3	[82]
Grape	SPE	HPLC-MS/MS	60	190	[83]
Fruits and Vegetables	SPE	HPLC-MS/MS	1.2	5	[84]
Guava	SPE	CE-ECL	10	nd	[85]
Wheat	SPE followed by derivatization	FASI-MEKC	30	100	[86]
Rice Corn	Centrifugation with a mixture of water and acidified methanol at 1% (1:1)	HPLC-MS/MS	2 4	10	[87]
Cereals	Ultrasonication with water	HPLC-MS/MS	20	nd	[88]
Soy Corn	SPE	HPLC-MS/MS	140 150	420 450	[89]
Oil	Centrifugation with acidified water at 1%	HPLC-MS/MS	3.3	10	[43]
Beer	SPE	HPLC-MS/MS	0.2	0.5	[90]
Several aliments Several beverages	SPE	HPLC-MS/MS	0.3 0.2	1 0.5	[47]
Several aliments	SPE followed by derivatization	HPLC-MS/MS	1.7	5	[91]

CE-ECL—Capillary Electrophoresis with Electrochemiluminescence. FASI-MEKC—Field-Amplified Sample Injection and Sweeping Micellar Electrokinetic Chromatography. FI-MS/MS—Flow Injection with tandem Mass Spectrometry. HPLC-MS/MS—High Performance Liquid Chromatography-tandem Spectrometry Mass. IC-HRMS—Ion Chromatography with tandem High Resolution Mass Spectrometry. IC-MS/MS—Ion Chromatography with tandem Mass Spectrometry. nd—Not defined. SPE—Solid Phase Extraction. UHPLC-MS/MS—Ultra High Performance Liquid Chromatography with tandem Mass Spectrometry.

**Table 4:** Occurrence of glyphosate in honey.

Country	Number of Samples	Detection Frequency (%)	Minimum (μg/kg)	Mean (μg/kg)	Maximum (μg/kg)	References
Canada	200	98.5	1	4.9	49.8	[19]
Switzerland	16	93.8	<1	4.6	15.9	[47]
Estonia	33	12.1	9	35	62	[79]
USA	85	28.2	15	92.4	342	[92]
Several European Countries	186	12.9	nd	nd	nd	[93]

nd—not determined.

**Table 5:** Occurrence of glyphosate in fruits and nuts.

Country	Matrix	Number of Samples	Detection Frequency (%)	Minimum (μg/kg)	Mean (μg/kg)	Maximum (μg/kg)	References
Switzerland	Fruit juice	11	100	0.5	1.9	3.5	[47]
France	Fruit	6	0	<5	<5	<5	[91]
China	Fruit	15	6.7	20	20	20	[84]
Several European Countries	Pear	627	1.0	nd	nd	nd	[93]
	Orange	625	0.8	nd	nd	nd	
	Apple	340	0.3	nd	nd	nd	
	Strawberry	308	0.3	nd	nd	nd	
	Blackberry	68	4.4	nd	nd	nd	
	Lime	58	5.2	nd	nd	nd	
	Raisin	48	2.1	nd	nd	nd	
	Walnut	14	7.1	nd	nd	nd	
Portugal	Orange	11	0	<100	<100	<100	[94]
	Pear	13	0	<100	<100	<100	

nd—not determined.

**Table 6:** Occurrence of glyphosate in cereals and cereal products.

Country	Matrix	Number of Samples	Detection Frequency (%)	Minimum (μg/kg)	Mean (μg/kg)	Maximum (μg/kg)	References
Switzerland	Breakfast Cereals	10	80	<1	50.8	291	[47]
	Wheat	18	88.9	<1	134.9	421	
	Snacks	11	36.4	<1	3.7	17.9	
	Bread	10	70	<1	6.9	45.8	
	Wheat Flower	28	28.6	<1	10.6	133	
	Pseudo cereals	3	0	<1	<1	<1	
	Other cereals	13	15.4	<1	1.2	12.4	
Italy	Wheat flower	4	0	<30	<30	<30	[86]
	Wheat seeds	1	100	243,000	243,000	243,000	
France	Breakfast Cereals	2	100	6	20	34	[91]
Several European Countries	Wheat	676	9.0	nd	nd	nd	[93]
	Rye	534	3.4	nd	nd	nd	
	Rice	266	0.4	nd	nd	nd	
	Oat	61	4.9	nd	nd	nd	
	Barley	51	23.5	nd	nd	nd	
	Linseeds	48	16.7	nd	nd	nd	
	Pseudo cereals	45	8.9	nd	nd	nd	

nd—not determined.

**Table 7:** Occurrence of glyphosate in vegetables and pulses.

Country	Matrix	Number of Samples	Detection Frequency (%)	Minimum (μg/kg)	Mean (μg/kg)	Maximum (μg/kg)	References
Ghana	Yam	68	20.5	<120	<120	<120	[13]
Switzerland	Potato and vegetables	10	30	<1	1.3	7.7	[47]
France	Vegetables	14	0	<5	<5	<5	[91]
Italy	Vegetables	83	18.1	3	nd	300	[82]
China	Vegetables	35	0	<5	<5	<5	[84]
Several European Countries	Asparagus	319	0.9	nd	nd	nd	[93]
	Pepper	215	0.5	nd	nd	nd	
	Peas	20	25	nd	nd	nd	
Switzerland	Pulses	41	51.2	<1	173.3	2948	[47]
Several European Countries	Dried Lentils	79	41.8	nd	nd	nd	[93]

nd—not determined.

**Table 8:** Occurrence of glyphosate in animal products.

Country	Matrix	Number of Samples	Detection Frequency (%)	Minimum (μg/kg)	Mean (μg/kg)	Maximum (μg/kg)	References
Switzerland	Milk	3	0	<0.5	<0.5	<0.5	[47]
	Egg	1	0	<1	<1	<1	
	Meat and Fish	13	23.1	<1	0.8	4.9	

**Table 9:** Occurrence of glyphosate in baby food.

Country	Matrix	Number of Samples	Detection Frequency (%)	Minimum (μg/kg)	Mean (μg/kg)	Maximum (μg/kg)	References
Switzerland	Baby food	11	0	<1	<1	<1	[47]
France	Baby food	71	0	<2	<2	<2	[91]
Italy	Baby food	15	13.3	3	3	3	[82]

**Table 10:** Occurrence of glyphosate in water.

Country	Matrix	Number of Samples	Detection Frequency (%)	Minimum (μg/L)	Mean (μg/L)	Maximum (μg/L)	References
Switzerland	Surface water	151	0	<0.02	<0.02	<0.02	[95]
Mexico	Groundwater	29	89.7	<0.05	0.94	1.70	[96]
	Bottled drinking water	15	86.7	<0.05	0.48	0.78	
Germany	Surface water	39	59	<0.025	0.12	0.59	[97]
USA	Several types of water	3732	39.4	<0.02	nd	476	[98]
Several European Countries	Surface water	50,805	28.9	<0.003	nd	50	[99]
	Groundwater	36,298	1.3	<0.01	nd	24	

nd—not determined.

**Table 11:** Occurrence of glyphosate in alcoholic beverages.

Country	Matrix	Number of Samples	Detection Frequency (%)	Minimum (μg/L)	Mean (μg/L)	Maximum (μg/L)	References
Switzerland	Wine	21	100	0.6	4.8	18.9	[47]
	Beer	15	13.3	<0.5	0.6	6.8	
Latvia	Beer	100	92	<0.5	7.5	150	[90]

## Supplementary Materials

The following are available online at https://www.mdpi.com/article/10.3390/foods10112785/s1, Table S1: Herbicides sales, in tons, in the EU between 2011 and 2018, Table S2: Herbicide applied per hectare of farmland in the EU between 2011 and 2018, Table S3: Glyphosate sales, in tons, in some European countries between 2011 and 2017, Table S4: Glyphosate applied per hectare of farmland in some European countries between 2011 and 2017, Table S5: Maximum residue levels in food.
