# Supplementary material for: Glyphosate Use, Toxicity and Occurrence in Food
Source: Foods. 2021 Nov 12;10(11):2785. doi: 10.3390/foods10112785 (PMC8622992; doi:10.3390/foods10112785)
Supplement: Supplementary file 1 [file foods-10-02785-s001.zip › foods-1440874-supplementary.pdf]

Supporting information for: Glyphosate use, toxicity and occurrence in food

Diogo A.F. Soares [a], Liliana J.G. Silva [a], Sofia C. Duarte [a,b], Angelina Pena [a], André M.P.T. Pereira [a,*]

[a] *LAQV, REQUIMTE, Group of Bromatology and Pharmacognosy, Faculty of Pharmacy, University of Coimbra, Polo III, Azinhaga de St[a] Comba, 3000-548 Coimbra, Portugal*

[b] *Department of Veterinary Sciences, Vasco da Gama Research Center, Vasco da Gama University School, 3020-210, Coimbra, Portugal*

***Corresponding author:**

LAQV, REQUIMTE, Group of Bromatology, Pharmacognosy and Analytical Sciences, Faculty of Pharmacy, University of Coimbra

Pólo das Ciências da Saúde, Azinhaga de Santa Comba, 3000-548 Coimbra, Portugal.

Tel: +351239488400

Fax : +351239827126

E-mail addresses:

daniguito97@hotmail.com (D.A.F. Soares), ljgsilva@hotmail.com (L.J.G. Silva), apena@ci.uc.pt (A. Pena), amptpereira@gmail.com (A.M.P.T. Pereira)

Table S1. Herbicides sales, in tones, in the EU between 2011 and 2018 [1].

Country	2011	2012	2013	2014	2015	2016	2017	2018
**Belgium**	2 482,59	2 720,22	2 486,43	2 519,65	2 372,95	2 261,41	2 334,15	2 648,07
**Bulgaria**	NA	773,569	705,944	652,446	636,209	2 007,43	1 698,98	2 606,57
**Czech Republic**	3 473,32	3 606,88	3 144,89	2 755,34	2 889,38	2 892,77	2 562,12	2 574,27
**Denmark**	3 692,16	4 563,85	2 935,90	1 238,59	1 903,40	1 914,30	1 904,68	NA
**Germany**	17 955,17	19 907,27	17 896,28	17 876,68	16 327,25	15 038,22	16 706,25	14 532,75
**Estonia**	357,09	436,89	434,25	425,85	472,28	604,15	462,64	428,20
**Ireland**	2 831,04	1 996,15	2 004,50	2 039,24	2 098,04	2 243,47	1 822,91	1 833,05
**Greece**	1 454,63	2 139,11	2 571,54	1 194,61	1 315,13	1 744,04	1 673,68	1 833,05
**Spain**	13 834,60	13 984,90	14 719,76	14 908,03	15 586,63	15 224,45	16 077,36	16 592,91
**France**	29 252,30	27 800,65	27 882,96	31 034,70	30 595,77	30 155,58	30 252,65	34 392,26
**Croatia**	NA	NA	829,44	889,12	820,90	742,56	668,74	717,67
**Italy**	8 327,29	8 055,92	7 750,99	7 798,75	7 950,43	7 486,50	7 114,26	6 880,13
**Cyprus**	169,99	137,00	127,68	158,41	116,84	157,65	138,93	160,58
**Latvia**	722,05	789,14	728,07	847,47	861,08	986,89	801,18	965,29
**Lithuania**	1 772,87	1 715,22	1 421,92	1 394,24	1 371,54	1 432,73	1 251,55	1 053,60
**Luxembourg**	102,07	96,47	82,78	88,60	83,35	61,71	60,77	54,18
**Hungary**	3 668,07	3,824,104	3 562,13	4 011,14	4 270,18	4 580,32	4 269,85	3 824,09
**Malta**	6,22	7,96	7,01	7,63	4,75	5,61	2,24	3,25

**The Netherlands**	3 024,61	3 042,16	2 766,24	3 266,27	2 880,90	2 745,75	2 901,79	2 978,20
**Austria**	1 505,16	1 544,52	1 227,02	1 375,82	1 317,22	1 281,49	1 296,94	1 276,93
**Poland**	12 408,49	12 654,36	12 518,20	12 073,41	12 190,01	12 693,32	13 655,48	11 370,74
**Portugal**	1 995,99	1 768,62	1 611,02	2 410,80	2 122,47	1 905,18	1 899,47	1 939,90
**Romania**	6 770,90	6 614,04	6 034,25	5 025,37	6 353,16	5 066,29	5 486,48	5 187,91
**Slovenia**	264,29	257,01	223,47	238,50	224,43	247,00	235,30	256,84
**Slovakia**	1 079,87	1 257,38	1 157,48	1 215,10	1 218,22	1 080,28	1 105,10	1 328,92
**Finland**	1 452,13	1 223,84	1 132,95	1 305,39	1 367,91	1 264,47	1 006,41	982,36
**Sweden**	2 136,11	2 087,12	3 545,63	2 103,77	1 829,32	1 633,01	1 731,24	1 482,79

NA, not available

Table S2. Herbicide applied per hectare of farmland in the EU between 2011 and 2018 [1,2].

Country	Farmland (ha)	Herbicide sales in the EU (kg)		Herbicide applied per farmland (kg/ha)	
		2011	2018	2011	2018
**Germany**	16 715 000	17 955 168	14 532 748	1,07	0,87
**Austria**	2 670 000	1 505 163	1 276 929	0,56	0,48
**Belgium**	1 354 000	2 482 593	2 648 068	1,83	1,96
**Bulgaria**	4 469 000	NA	2 606 572	NA	0,58
**Cyprus**	112 000	169 986	160 580	1,52	1,43
**Croatia**	1 563 000	NA	717 673	NA	0,46
**Denmark**	2 615 000	3 692 158	NA	1,41	NA
**Slovakia**	1 890 000	1 079 871	1 328 923	0,57	0,70
**Slovenia**	488 000	264 289	256 840	0,54	0,53
**Spain**	23 230 000	13 834 600	16 592 907	0,60	0,71
**Estonia**	995 000	357 091	428 200	0,36	0,43
**Finland**	2 194 000	1 452 126	982 359	0,66	0,45
**France**	27 814 000	29 252 304	34 392 257	1,05	1,24
**Greece**	4 554 000	1 454 632	1 833 052	0,32	0,40
**Hungary**	4 671 000	3 668 067	3 824 089	0,79	0,82

**Ireland**	4 884 000	2 831 037	1 833 054	0,58	0,38
**Italy**	12 598 000	8 327 293	6 880 130	0,66	0,55
**Latvia**	1 931 000	722 050	965 291	0,37	0,50
**Lithuania**	2 925 000	1 772 873	1 053 599	0,61	0,36
**Luxembourg**	131 000	102 073	54 178	0,78	0,41
**Malta**	11 000	6 223	3 247	0,57	0,30
**The Netherlands**	1 796 000	3 024 613	2 978 199	1,68	1,66
**Poland**	14 406 000	12 408 486	11 370 744	0,86	0,79
**Portugal**	3 642 000	1 995 991	1 939 900	0,55	0,53
**Czech Republic**	3 455 000	3 473 321	2 574 269	1,01	0,75
**Romania**	12 503 000	6 770 904	5 187 911	0,54	0,41
**Sweden**	3 021 000	2 136 107	1 482 790	0,71	0,49

NA, not available

Table S3. Glyphosate sales, in tones, in some European countries between 2011 and 2017 [3,4,13–21,5–12].

Country	2011	2012	2013	2014	2015	2016	2017
**France**	8 463,00	9 062,00	8 673,00	9 488,00	8 469,00	8 721,00	8 631,00
**Germany**	5 400,00	6 000,00	5 100,00	5 400,00	4 300,00	3 800,00	4 700,00
**Denmark**	1 941,31	1 402,52	1 388,86	626,84	853,75	1 140,70	1 241,40
**Portugal**	1 378,08	1 131,92	1 040,43	1 687,49	1 485,72	1 306,64	1 302,34
**Czech Republic**	962,61	1 081,32	935,47	858,51	697,69	772,33	750,53
**Belgium**	552,86	699,39	587,04	595,59	511,65	503,27	619,30
**Estonia**	227,90	292,06	c	276,97	c	411,61	NA

c, confidential; NA, not available

Table S4. Glyphosate applied per hectare of farmland in some European countries between 2011 and 2017 [2–6,14,20,21].

Country	Farmland	Glyphosate sales (kg)		Glyphosate applied per farmland (kg/ha)	
		2011	2017	2011	2017
**France**	27 814 000	8 463 000	8 631 000	0,30	0,31
**Germany**	16 715 000	5 400 000	4 700 000	0,32	0,28
**Denmark**	2 615 000	1 941 310	1 241 402	0,74	0,47
**Portugal**	3 642 000	1 378 081	1 302 344	0,38	0,36
**Czech Republic**	3 455 000	962 612	750 531	0,28	0,22
**Belgium**	1 354 000	552 861	619 295	0,41	0,46
**Estonia**	995 000	227 902	NA	0,23	NA

NA, not available

Table S5 – Maximum residue levels in food [22].

MATRIX	MRL (µg/kg)
**FRUITS**	
**Fruit juice**	10
**Pear**	100
**Orange**	500
**Apple**	100
**Strawberry**	100
**Blueberry**	100
**Lime**	100
**Raisin**	100
**Nut**	100
**Other fruits**	100
**VEGETABLES**	
**Yam**	100
**Potato**	500
**Asparagus**	100
**Pepper**	100
**Fresh peas**	100
**Other vegetables**	100
**PULSES**	
**Dry peas**	10 000
**Beans**	2 000
**Dry lentils**	10 000
**Other pulses**	10 000

MATRIX	MRL (µg/kg)
**CEREALS**	
**Barley**	20 000
**Pseudo cereals**	100
**Corn**	10 000
**Oat**	20 000
**Rice**	100
**Rye**	10 000
**Wheat**	10 000
**Other Cereals**	100
**ANIMAL PRODUCTS**	
**Egg**	50
**Meat**	50
**Fish**	50
**Milk**	50
**Honey**	50
**OTHERS**	
**Bread**	10
**Oil**	100
**Olive Oil**	100
**Water**	0,1[a]
**Baby food**	10

[a], MRL units for water are in µg/L

References:

1. Eurostat Sales of pesticides by type of pesticides Available online: https://appsso.eurostat.ec.europa.eu/nui/submitViewTableAction.do.

2. Eurostat *Agriculture, forestry and fishery statistics - 2018 Edition*; Publications Office of the European Union: Luxembourg:, 2018; ISBN 9789279947582.

3. Janson, M. So viel Glyphosat kommt in Deutschland zum Einsatz.

4. SPF Santé publique Sécurité de la Chaîne Alimentaire et Environnement Données de vente des produits phytopharmaceutiques en Belgique.

5. Rebane, R.; Vooro, K.; Nurk, G.; Leisk, Ü.; Laht, M.; Metsur, M. *Uuring pestitsiidide koormuse allikate ja päritolu selgitamiseks nitraaditundlikul alal*; Tallinn, 2017;

6. Ørum, J.E.; Martensen, K.Ø. *Bekæmpelsesmiddelstatistik 2017: Behandlingshyppighed og pesticidbelastning baseret på salg og forbrug*; København, 2019; ISBN 9788770380539.

7. Musil, B. Czech Republic - Usage of active substances in 2011 Available online: http://eagri.cz/public/web/file/439602/celek_2011_EN.pdf.

8. Musil, B. Czech Republic - Usage of active substances in 2012 Available online: http://eagri.cz/public/web/file/439598/celek_2012_EN.pdf.

9. Musil, B. Czech Republic - Usage of active substances in 2013 Available online: http://eagri.cz/public/web/file/439536/celek_2013_EN.pdf.

10. Musil, B. Czech Republic - Usage of active substances in 2014 Available online: http://eagri.cz/public/web/file/439456/celek_2014_EN.pdf.

11. Musil, B. Czech Republic - Usage of active substances in 2015 Available online: http://eagri.cz/public/web/file/477476/Spotreba_UL_2015_EN_CELEK.pdf.

12. Musil, B. Czech Republic - Usage of active substances in 2016 Available online: http://eagri.cz/public/web/file/537723/celek_2016_EN.pdf.

13. Musil, B. Czech Republic - Usage of active substances in 2017 Available online: http://eagri.cz/public/web/file/587988/celek_2017_EN.pdf.

14. Direção Geral de Alimentação e Veterinára Vendas de Produtos Fitofarmacêuticos em Portugal em 2011.

15. Direção Geral de Alimentação e Veterinára *Vendas de Produtos Fitofarmacêuticos em Portugal em 2012*; Lisboa, 2013;

16. Direção Geral de Alimentação e Veterinára *Vendas de Produtos Fitofarmacêuticos em Portugal em 2013*; Lisboa, 2015;

17. Direção Geral de Alimentação e Veterinára *Vendas de Produtos Fitofarmacêuticos em Portugal em 2014*; Lisboa, 2016;

18. Direção Geral de Alimentação e Veterinára Vendas de Produtos Fitofarmacêuticos em Portugal em 2015 Available online: https://www.dgav.pt/wp-content/uploads/2021/05/Vendas-de-Produtos-Fitofarmaceuticos-Portugal-2015.pdf.

19. Direção Geral de Alimentação e Veterinára Vendas de Produtos Fitofarmacêuticos em Portugal em 2016 Available online: https://www.dgav.pt/wp-content/uploads/2021/05/Relatorio-Vendas-2016_PF.pdf.

20. Direção Geral de Alimentação e Veterinára Vendas de Produtos Fitofarmacêuticos em Portugal em 2017 Available online: https://www.agroportal.pt/wp-content/uploads/vendas-2017.pdf.

21. Commissariat général au développement durable Plan de réduction des produits phytopharmaceutiques et sortie du glyphosate : état des lieux des ventes et des achats en France. *DATALAB Essent.* **2019**, *172*, 4.

22. European Comission EU Pesticides database - Pesticides residues and maximum residue levels.
